# Silenciar ou Estabilizar: Eis a Questão

**DOI:** 10.36660/abc.20250426

**Published:** 2025-09-10

**Authors:** João Bicho Augusto

**Affiliations:** 1 Hospital Prof. Dr. Fernando Fonseca Serviço de Cardiologia Amadora Portugal Serviço de Cardiologia, Hospital Prof. Dr. Fernando Fonseca, Amadora – Portugal; 2 Hospital CUF Tejo Centro de Coração e Vasos Lisboa Portugal Centro de Coração e Vasos, Hospital CUF Tejo, Lisboa – Portugal; 3 Católica Medical School Lisboa Portugal Católica Medical School, Lisboa – Portugal; 4 University College London Institute of Cardiovascular Science Londres Reino Unido Institute of Cardiovascular Science, University College London, Londres – Reino Unido

**Keywords:** Amiloidose, Transtirretina, Cardiomiopatia

A cardiomiopatia por transtirretina amiloide (ATTR-CM) era anteriormente considerada uma doença progressiva inevitável, com desfechos devastadores. Os médicos frequentemente a desconsideravam, visto que o arsenal terapêutico disponível era muito limitado. Historicamente, os tratamentos ofereciam principalmente alívio sintomático, sem alterar a progressão da doença. Felizmente, a última década nos trouxe tratamentos modificadores da doença com o potencial de transformar o curso da ATTR-CM.^1^

Em nível molecular, a ATTR-CM se desenvolve quando a transtirretina (TTR), geralmente uma proteína transportadora estável para tiroxina e vitamina A, se dissocia de sua configuração tetramérica em monômeros e fibrilas amiloides que se acumulam no miocárdio.^2^ Duas classes principais de fármacos têm sido estudadas para o tratamento da ATTR-CM: estabilizadores e silenciadores. Estabilizadores, como tafamidis e acoramidis, atuam estabilizando a configuração tetrâmera da TTR, prevenindo assim sua degradação e subsequente deposição de fibrilas amiloides.^3 , 4^ Em contraste, silenciadores, como patisiran e vutrisiran, inibem a produção hepática de TTR por meio de mecanismos de interferência de RNA, reduzindo essencialmente a fonte do precursor amiloide.^5 , 6^ Historicamente, o estudo ATTR-ACT foi o primeiro ensaio de Fase III a demonstrar que um medicamento para ATTR-CM, o tafamidis, poderia reduzir a mortalidade por todas as causas, dando aos estabilizadores uma vantagem competitiva.^3^ No entanto, com classes de medicamentos mais novas, qual opção é a melhor para nossos pacientes?

Para esclarecer e (indiretamente) comparar a eficácia dessas estratégias, Facin et al. conduziram uma revisão sistemática abrangente e meta-análise abrangendo sete ensaios clínicos randomizados (ECRs) envolvendo mais de 2.500 pacientes com ATTR-CM.^7^ Em todos os ensaios, estabilizadores ou silenciadores foram comparados com placebo. Os autores descobriram que os estabilizadores de TTR diminuíram significativamente a mortalidade e as hospitalizações, fornecendo fortes evidências em apoio ao seu uso clínico. Em contraste, os silenciadores não mostraram nenhum impacto significativo nesses resultados primários; no entanto, eles melhoraram a capacidade funcional, a qualidade de vida (por exemplo, distância do teste de caminhada de seis minutos) e os níveis de biomarcadores (por exemplo, NT-proBNP).^7^ Esses resultados lançam luz sobre o cenário atual do tratamento de ATTR-CM, mas não são uma comparação direta. Essa aparente diferença entre classes de medicamentos deve ser interpretada com base em evidências acumuladas e experiência do mundo real.

Em primeiro lugar, as populações de pacientes diferem significativamente entre o estudo ATTR-ACT e os ensaios subsequentes, visto que o primeiro incluiu pacientes com doença mais grave (uma vez que nenhum medicamento eficaz havia sido aprovado na época, os diagnósticos eram escassos e tipicamente tardios) e menos formas de TTR selvagem (versus TTR hereditária).^3 , 8^ Desde então, a conscientização sobre o ATTR-CM aumentou e os métodos de diagnóstico melhoraram, também motivados pela disponibilidade do tafamidis, que permite a detecção precoce da doença. O resultado é uma mudança necessária nas características basais dos pacientes em estudos subsequentes. De modo geral, comparações diretas entre estabilizadores e silenciadores não são possíveis devido à discrepância entre as populações do estudo incluídas nos ECRs correspondentes.^7 , 9^

Em segundo lugar, os ECRs com silenciadores TTR tiveram duração tipicamente mais curta, limitando o acúmulo de eventos clínicos e o poder estatístico.^5 , 6^ Curiosamente, dados recentes de acompanhamento prolongado do estudo HELIOS-B (não incluído nesta meta-análise no momento da revisão por pares do manuscrito) revelaram benefícios significativos do vutrisiran (um silenciador TTR), com reduções na mortalidade por todas as causas, mortalidade cardiovascular, hospitalizações e admissões por insuficiência cardíaca.^6^ A exclusão deste conjunto de dados desta meta-análise introduz um viés contra silenciadores. O potencial benefício clínico dos silenciadores permanece promissor, destacando a necessidade de períodos mais longos de acompanhamento.^7^

Em terceiro lugar, como os pacientes agora são diagnosticados e tratados mais precocemente, estudos recentes (como APOLLO-B e HELIOS-B) incluíram pacientes com estágios mais leves de ATTR-CM.^5 , 6^ Essas distinções são importantes, pois influenciam as taxas de eventos, a resposta à terapia e, em última análise, nossa avaliação do benefício do tratamento. Deve-se notar que os silenciadores oferecem uma via biológica distinta, visando a formação de amiloide, o que pode ter implicações para o tratamento da doença em estágio inicial ou mesmo pré-sintomática, onde a redução da TTR circulante pode ter o potencial de interromper completamente a deposição de amiloide.^5 , 6^ Mais ECRs são necessários para abordar essa questão.

Finalmente, ECRs recentes adicionaram camadas de complexidade ao incluir pacientes em terapia combinada. O conceito de usar um silenciador e um estabilizador juntos é biologicamente atraente.^6 , 9^ No entanto, as evidências ainda são escassas, e não podemos ignorar os custos subjacentes para os sistemas de saúde. As avaliações de pacientes já em tafamidis no HELIOS-B são observacionais em vez de randomizadas e são influenciadas pelo viés de seleção.^6^ O grande estudo CARDIO-TTRansform em andamento visa estudar eplontersen (silenciador TTR) em mais de 1.400 pacientes, o que deve lançar luz sobre o uso de silenciadores TTR como terapia de primeira linha e esclarecer o uso da terapia combinada em ATTR-CM.^10^ Até que tenhamos evidências robustas, a estratégia de combinação deve ser reservada para subgrupos específicos de pacientes, como aqueles que apresentam falha do tratamento.^9^

Agora temos opções em ATTR-CM, mas ainda enfrentamos um dilema: devemos priorizar estabilizadores com benefícios comprovados em termos de mortalidade ou explorar as vantagens recentemente sugeridas dos silenciadores? Além disso, quem são os pacientes que mais podem se beneficiar dos silenciadores?

Embora os estabilizadores pareçam atualmente ser a terapia de primeira linha para ATTR-CM, os silenciadores podem ser pelo menos tão eficazes em subgrupos específicos de pacientes, particularmente em casos de TTR hereditária, doença inicial (com fração de ejeção do ventrículo esquerdo preservada), polineuropatia concomitante ou em casos com contraindicações aos estabilizadores^5 , 6 , 9^ – ver Tabela 1 . Esta meta-análise representa um progresso substancial e abre caminho para o tratamento personalizado. A escolha de uma classe medicamentosa em detrimento de outra é atualmente uma estratégia que depende de uma panóplia de fatores e inclui as evidências que temos acumulado nos últimos anos. Embora um algoritmo de tratamento seja preferível, esse "desconforto clínico" é essencialmente a arte da Medicina.

**Tabela 1:** Comparação de estabilizadores e silenciadores TTR no tratamento ATTR-CM

Recurso	Estabilizadores TTR	Silenciadores TTR
Mecanismo de ação	Estabilizar o tetrâmero TTR, prevenindo a dissociação em fibrilas amiloides	Reduzir a produção hepática de TTR (interferência de RNA)
Exemplos de drogas	Tafamidis, Acoramidis	Patisiran, Vutrisiran, Inotersen
Impacto na mortalidade	Redução significativa estabelecida (ATTR-ACT, ATTRibute)	Redução promissora e significativa no acompanhamento prolongado (HELIOS-B)
Redução de hospitalizações	Benefício significativo comprovado	Evidências promissoras (HELIOS-B estendido)
Melhoria do estado funcional	Melhoria significativa	Melhoria significativa
Biomarcadores (por exemplo, NT-proBNP)	Redução estabelecida	Redução significativa estabelecida
Perfis de pacientes ideais	ATTR-CM estabelecido, doença mais grave	ATTR hereditário, ATTR-CM em estágio inicial, função do ventrículo esquerdo preservada, polineuropatia
Considerações renais/hepáticas	Metabolismo hepático (tafamidis); cautela em casos de disfunção hepática grave, dados limitados em casos de insuficiência renal grave	Geralmente favorável, particularmente vutrisiran (perfil hepático e renal seguro)

TTR: transtirretina; ATTR: transtirretina amiloide; ATTR-CM: cardiomiopatia por transtirretina amiloide.
