# Supplementary material for: Silenciar ou Estabilizar: Eis a Questão
Source: Arq Bras Cardiol. 2025 Sep 10;122(8):e20250426. [Article in Portuguese] doi: 10.36660/abc.20250426 (PMC12671724; doi:10.36660/abc.20250426)
Supplement: Translation

# Translation: To Silence or to Stabilize: That is the Question

**DOI:** 10.36660/abc.20250426i

**Authors:** João Bicho Augusto

**Affiliations:** 1 Amadora Portugal Serviço de Cardiologia, Hospital Prof. Dr. Fernando Fonseca, Amadora – Portugal; 2 Lisboa Portugal Centro de Coração e Vasos, Hospital CUF Tejo, Lisboa – Portugal; 3 Lisboa Portugal Católica Medical School, Lisboa – Portugal; 4 Londres Reino Unido Institute of Cardiovascular Science, University College London, Londres – Reino Unido

**Keywords:** Amyloidosis, Transthyretin, Cardiomyopathy

Transthyretin amyloid cardiomyopathy (ATTR-CM) was previously considered an inescapable progressive disease leading to devastating outcomes. Clinicians often disregarded ATTR-CM, as the therapeutical arsenal available was very limited. Historically, treatments have offered mostly symptomatic relief without altering disease progression. Fortunately, the last decade brought us disease-modifying treatments with the potential to transform the course of ATTR-CM.^1^

At a molecular level, ATTR-CM develops when transthyretin (TTR), usually a stable transport protein for thyroxine and vitamin A, dissociates from its tetrameric configuration into monomers and amyloid fibrils that accumulate in the myocardium.^2^Two key drug classes have been studied for the treatment of ATTR-CM: stabilizers and silencers. Stabilizers, such as tafamidis and acoramidis, act by stabilizing the TTR tetramer configuration, thus preventing its breakdown and subsequent deposition of amyloid fibrils.^3 , 4^In contrast, silencers, such as patisiran and vutrisiran, inhibit the hepatic production of TTR via RNA interference mechanisms, essentially reducing the source of the amyloid precursor.^5 , 6^Historically, the ATTR-ACT study was the first Phase III trial to demonstrate that a drug for ATTR-CM, tafamidis, could reduce all-cause mortality, giving stabilizers a competitive edge.^3^However, with newer drug classes, which option is the best for our patients?

To clarify and (indirectly) compare the effectiveness of these strategies, Facin et al. conducted a comprehensive systematic review and meta-analysis encompassing seven randomized controlled trials (RCTs) involving over 2,500 ATTR-CM patients.^7^In all trials, either stabilizers or silencers were compared against placebo. The authors found that TTR stabilizers significantly decreased mortality and hospitalizations, providing strong evidence in support of their clinical use. In contrast, silencers did not show any significant impact on these primary outcomes; however, they did improve functional capacity, quality of life (e.g., six-minute walk test distance), and biomarker levels (e.g., NT-proBNP).^7^These results shed light on the current landscape of ATTR-CM treatment, but they are not a head-to-head comparison. Such a seeming difference between drug classes must be interpreted based on accumulating evidence and real-world experience.

First, patient populations differ significantly between the ATTR-ACT study and subsequent trials, as the former included patients with more severe disease (since no efficient drug was approved at the time, diagnoses were scarce and typically late) and fewer forms of wild-type TTR (versus hereditary TTR).^3 , 8^Since then, ATTR-CM awareness has increased, and diagnostic methods have improved, also motivated by the availability of tafamidis, which allows for earlier disease detection. The result is a necessary change of patient baseline characteristics in subsequent studies. Overall, direct comparisons between stabilizers and silencers are not possible due to the discrepancy between the study populations included in the corresponding RCTs.^7 , 9^

Second, RCTs of TTR silencers were typically of shorter duration, limiting the accumulation of clinical events and statistical power.^5 , 6^Interestingly, recent extended follow-up data from the HELIOS-B trial (not included in this meta-analysis at the time the manuscript was peer-reviewed) revealed significant benefits of vutrisiran (a TTR silencer), with reductions in all-cause mortality, cardiovascular mortality, hospitalizations, and admissions for heart failure.^6^The exclusion of this dataset from this meta-analysis introduces a bias against silencers. The potential clinical benefit of silencers remains promising, highlighting the need for longer periods of follow-up.^7^

Third, since patients are now both diagnosed and treated earlier, recent trials (such as APOLLO-B and HELIOS-B) included patients with milder stages of ATTR-CM.^5 , 6^These distinctions are important as they influence event rates, response to therapy, and, ultimately, our assessment of treatment benefit. It should be noted that silencers offer a distinct biological pathway by targeting amyloid formation, which could have implications for the treatment of early or even pre-symptomatic disease, where reduction of circulating TTR could have the potential to halt amyloid deposition entirely.^5 , 6^More RCTs are needed to address this issue.

Finally, recent RCTs have added layers of complexity by including patients under combination therapy. The concept of using a silencer and a stabilizer together is biologically appealing.^6 , 9^However, evidence is still scarce, and we cannot overlook the underlying costs to the healthcare systems. Assessments of patients already on tafamidis in HELIOS-B are observational rather than randomized and are influenced by selection bias.^6^The ongoing, large CARDIO-TTRansform trial aims to study eplontersen (TTR silencer) in over 1,400 patients, which is expected to shed light on the use of TTR silencers as the first-line therapy and clarify the use of combination therapy in ATTR-CM.^10^Until we have robust evidence, the combination strategy should be reserved for specific patient subgroups, such as those who experience treatment failure.^9^

We now have choices in ATTR-CM, but we are still facing a dilemma: should we prioritize stabilizers with proven mortality benefits or explore the recently suggested advantages of silencers? Furthermore, who are the patients who might benefit the most from silencers?

Although stabilizers currently seem to be the first-line therapy for ATTR-CM, silencers might be at least as effective in specific subgroups of patients, particularly in hereditary TTR, early disease (with preserved left ventricular ejection fraction), concurrent polyneuropathy, or in cases with contraindications to stabilizers^5 , 6 , 9^– see Table 1 . This meta-analysis represents substantial progress and opens the window to personalized care. Choosing one drug class over the other is currently a strategy that depends on a panoply of factors and includes the evidence we have been accumulating in recent years. Although a treatment algorithm would be preferable, this "clinical discomfort" is essentially the art of Medicine.

**Table 1:** Comparação de estabilizadores e silenciadores TTR no tratamento ATTR-CM

Feature	TTR Stabilizers	TTR Silencers
Mechanism of action	Stabilize TTR tetramer, preventing dissociation into amyloid fibrils	Reduce hepatic TTR production (RNA interference)
Drugs examples	Tafamidis, Acoramidis	Patisiran, Vutrisiran, Inotersen
Mortality impact	Significant reduction established (ATTR-ACT, ATTRibute)	Promising, significant reduction in extended follow-up (HELIOS-B)
Hospitalizations reduction	Proven significant benefit	Promising evidence (extended HELIOS-B)
Functional status improvement	Significant improvement	Significant improvement
Biomarkers (e.g., NT-proBNP)	Reduction established	Significant reduction established
Ideal patient profiles	Established ATTR-CM, more severe disease	Hereditary ATTR, early-stage ATTR-CM, preserved left ventricular function, polyneuropathy
Renal/hepatic considerations	Hepatic metabolism (tafamidis); caution in severe hepatic dysfunction, limited data in severe renal impairment	Generally favorable, particularly vutrisiran (safe hepatic and renal profile)

TTR: transthyretin; ATTr: transthyretin amyloid; ATTR-CM: transthyretin amyloid cardiomyopathy.
